# Dynamic single cell analysis in a proximal-tubule-on-chip reveals heterogeneous epithelial colonization strategies of uropathogenic *Escherichia coli* under shear stress

**DOI:** 10.1093/femsmc/xtad007

**Published:** 2023-03-03

**Authors:** Haris Antypas, Tianqi Zhang, Ferdinand X Choong, Keira Melican, Agneta Richter-Dahlfors

**Affiliations:** AIMES – Center for the Advancement of Integrated Medical and Engineering Sciences, Karolinska Institutet and KTH Royal Institute of Technology, SE-171 77, Stockholm, Sweden; Department of Neuroscience, Karolinska Institutet, SE-171 77, Stockholm, Sweden; AIMES – Center for the Advancement of Integrated Medical and Engineering Sciences, Karolinska Institutet and KTH Royal Institute of Technology, SE-171 77, Stockholm, Sweden; Department of Neuroscience, Karolinska Institutet, SE-171 77, Stockholm, Sweden; AIMES – Center for the Advancement of Integrated Medical and Engineering Sciences, Karolinska Institutet and KTH Royal Institute of Technology, SE-171 77, Stockholm, Sweden; Department of Neuroscience, Karolinska Institutet, SE-171 77, Stockholm, Sweden; AIMES – Center for the Advancement of Integrated Medical and Engineering Sciences, Karolinska Institutet and KTH Royal Institute of Technology, SE-171 77, Stockholm, Sweden; Department of Neuroscience, Karolinska Institutet, SE-171 77, Stockholm, Sweden; AIMES – Center for the Advancement of Integrated Medical and Engineering Sciences, Karolinska Institutet and KTH Royal Institute of Technology, SE-171 77, Stockholm, Sweden; Department of Neuroscience, Karolinska Institutet, SE-171 77, Stockholm, Sweden

**Keywords:** optotracing, microfluidics, UPEC, biofilm, curli, microcolony

## Abstract

The urinary tract is a hydrodynamically challenging microenvironment and uropathogenic *Escherichia coli* (UPEC) must overcome several physiological challenges in order to adhere and establish a urinary tract infection. Our previous work *in vivo* revealed a synergy between different UPEC adhesion organelles, which facilitated effective colonization of the renal proximal tubule. To allow high-resolution real-time analysis of this colonization behavior, we established a biomimetic proximal-tubule-on-chip (PToC). The PToC allowed for single-cell resolution analysis of the first stages of bacterial interaction with host epithelial cells, under physiological flow. Time-lapse microscopy and single-cell trajectory analysis in the PToC revealed that while the majority of UPEC moved directly through the system, a minority population initiated heterogeneous adhesion, identified as either rolling or bound. Adhesion was predominantly transient and mediated by P pili at the earliest time-points. These bound bacteria initiated a founder population which rapidly divided, leading to 3D microcolonies. Within the first hours, the microcolonies did not express extracellular curli matrix, but rather were dependent on Type 1 fimbriae as the key element in the microcolony structure. Collectively, our results show the application of Organ-on-chip technology to address bacterial adhesion behaviors, demonstrating a well-orchestrated interplay and redundancy between adhesion organelles that enables UPEC to form microcolonies and persist under physiological shear stress.

## Teaser

Uropathogenic Escherichia coli use multiple adhesion strategies to facilitate colonisation and form microcolonies under flow

## Introduction

The ability of bacteria to adhere to tissue is essential to establishing infection. Bacteria attachment is heavily influenced by the physiology of the infection site and in many sites shear stress from fluid flow is an important factor. In the urinary tract, shear stress varies in the kidney as the body regulates renal function and changes dramatically upon bladder voiding. Our group has previously used a spatio-temporally controlled *in vivo* model of kidney infection to study the early stages of kidney colonization by uropathogenic *Escherichia coli* (UPEC) (Månsson et al. 2007). We identified a synergistic relationship between the important UPEC adhesion organelles P and Type 1 fimbriae (Melican et al. 2011). P fimbriae-mediated colonization of the proximal tubule epithelium during the first hours of infection under high shear stress, whereas Type 1 fimbriae enabled bacteria to colonize the tubule’s lumen away from the epithelium. Supported by previous data, this led us to hypothesize a major role for Type 1 fimbriae in mediating interbacterial binding and a “biofilm-like” lifestyle (Justice et al. 2004, 2006, Melican et al. 2011, Conover et al. 2016). Neither fimbriae were found to be essential to infection, but *in vivo* they influenced the kinetics of infection progression (Melican et al. 2011).

The complexity of *in vivo* models has prevented more mechanistic evaluation of this fimbrial synergy. To address this, we evaluated the usability of *in vitro* microfluidic technology known as “Organ-on-chip.” Organ-on-chip models are designed to recapitulate the physiology and/or the structure at organ and tissue level. Importantly for our work, these models include shear stress, which is often lacking from *in vitro* assays and is critical to bacterial binding *in vivo*. This technology has been widely adapted as biomimics of organs such as the liver, kidney, heart, lung, and intestine, but studies on host–pathogen interactions are relatively scarce.

In this work, we establish a “Proximal-tubule-on-chip” (PToC) platform to evaluate the initial binding and colonization characteristics of UPEC to renal epithelial cells under physiological shear stress conditions. We employ single cell tracking to describe distinct initial adhesion behaviors of UPEC in the PToC. We define the role for PapG in mediating initial adhesion behavior and adhesion duration. We show how FimH facilitates the formation of extensive 3D microcolonies, which are resistant to flow. These microcolonies do not express extracellular matrix (ECM) in the early hours of infection but rather are dependent on Type 1 fimbriae-mediated interbacterial binding. This work uses the PToC to describe the mechanistic and synergistic roles of these two important fimbriae in the initial colonization strategy of UPEC to renal epithelium, and demonstrates how they lead to effective epithelial colonization and microcolony formation during the first hours of infection under flow.

## Results

### Stationary and rolling adhesion of UPEC under shear stress in a PToC

**Figure 1.**
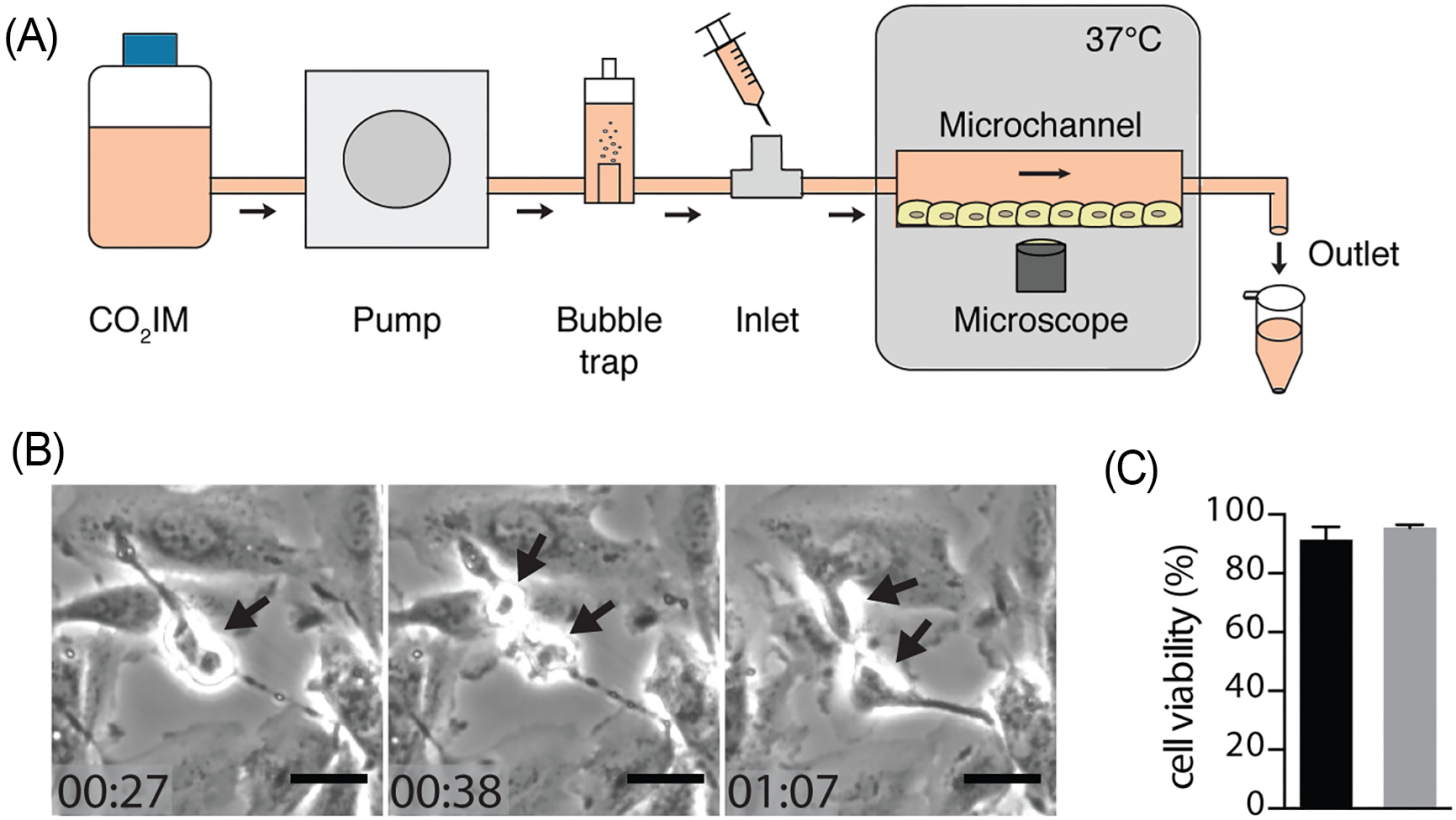
(A) Schematic representation of the PToC setup. A pump connected with tubing generates flow of CO_2_-independent medium (CO_2_IM) at a flow rate of 75 µl/min, equivalent to the primary filtrate flow rate in the S1 segment of the proximal tubule. Medium flows through a bubble trap, before entering the microchannels seeded with A498 cells. The slide is placed on the microscope stage at 37°C. Bacteria are introduced in the microchannel via the inlet and infection is monitored with phase-contrast time-lapse microscopy. Samples from the microchannel can be collected at the outlet. (B) Representative frames from a time-lapse video showing an A498 renal epithelial cell dividing in the PToC (black arrows). Scale bar = 15 µm, time = hh: mm. (C) Viability of A498 renal epithelial cells incubated in microchannels with CO_2_IM, under a flow rate of 75 μl/min (black) vs. static conditions (gray) for 8 h at 37°C. Average cell viability (%) from *n* = 2 is shown, error bar = SEM.

To study UPEC–host interactions under physiologically relevant conditions, we developed a PToC. This design features six microchannels, seeded with human renal epithelial cells (A498) (Fig. 1A). To mimic the conditions of the renal tubule in the microchannels, we mimicked the shear stress generated by the primary filtrate flow in the S1 proximal tubule, estimated to be 0.104 dyn/cm^2^ (Essig and Friedlander 2003). Renal epithelial cells were subjected to a flow of 75 µl/min of CO_2_ independent media (CO_2_IM). Under these conditions the cells exhibited healthy morphology, confluence, and proliferation, with a viability of 91.4 ± 6.05%, comparable to a 95.5 ± 2.9% viability under static conditions (Fig. 1B and C). To investigate how UPEC adheres to renal cells under shear stress, we used the UPEC strain CFT073 (wt) (Table S1, Supporting Information), which we confirmed expressed Type 1 and P fimbriae in CO_2_IM (Table S2, Supporting Information). Under flow, 0.5 ml of CFT073 (≈ 2 × 10^8^ cfu) was slowly infused via the inlet, over 1 min. To capture the modes of initial bacterial adhesion to renal cells, we performed time-lapse microscopy for 10 s at a high frame rate. We observed that the majority of bacteria were rapidly expelled by the flow, without interacting with cells, while other bacteria resisted the flow and were displaced at a lower speed (Movie 1).

**Figure 2.**
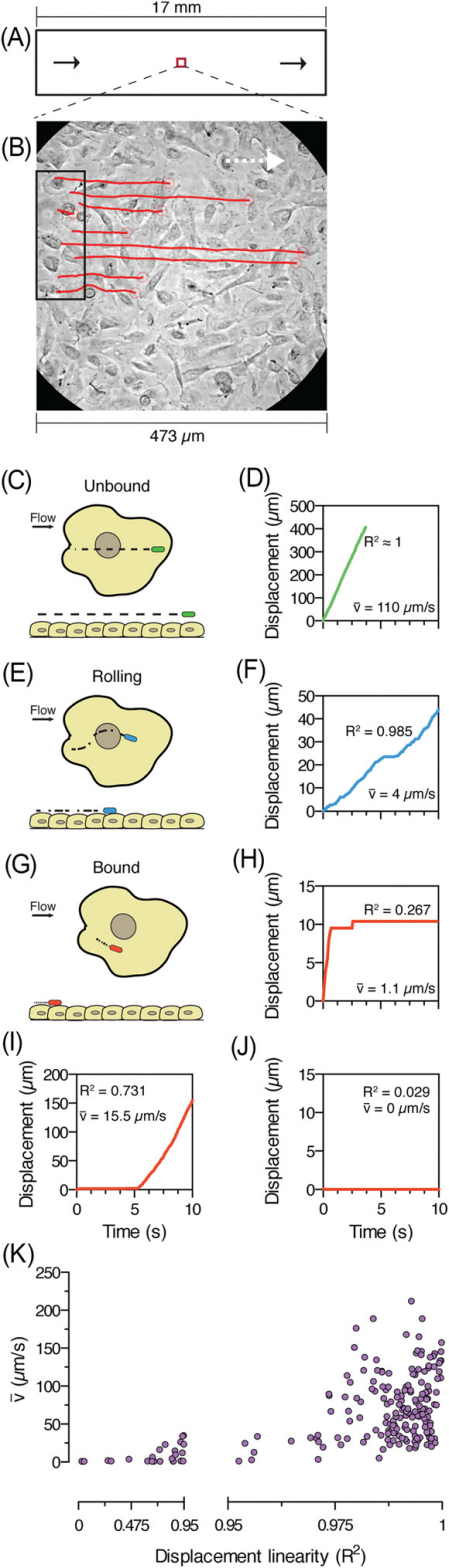
(A) Schematic representation of a microchannel’s top view. A microscopic field of view in the central lane of the microchannel is selected for time-lapse microscopy. Black arrows = flow direction (B) SCTA of bacteria in PToC. A starting area is selected (black box), and all bacteria found within this area on the first frame (*t* = 0 s) are followed until the last frame (*t* = 10 s). The trajectories of each bacterium throughout the time-lapse are connected by a red line. White arrow = flow direction. (C) Top and side view illustration of unbound bacteria’s trajectory (dashed line) under flow. The equally sized dashes of the trajectory show the constant displacement of bacteria over time. Side view demonstrates the absence of interactions between bacteria and cells. (D) Representative graph of total displacement over time from a single unbound bacterium. (E) Illustration of rolling bacteria’s trajectory (dash-dotted line) on renal cells under flow. The uneven dashes and dots of the trajectory show the fluctuating displacement over time. Side view shows bacterial interactions with cells. The shorter trajectory length compared to unbound bacteria indicates the lower velocity observed. (F) Representative graph of total displacement over time from a single rolling bacterium (G) Illustration of bound bacteria’s trajectory (dotted line) on renal cells under flow. The trajectory dots indicate that displacement is short and can be abruptly interrupted by stationary adhesion to the cell. Side view demonstrates that bacteria are interacting with cells and the very short trajectory length indicates the short distance traveled. (H)–(J) Total displacement over time from representative bound bacteria. Unbound bacteria could suddenly become bound (H) and vice versa (I), or be bound throughout the time-lapse recording (J). (K) Displacement linearity vs. mean velocity for individual CFT073 wt bacteria tracked from *n* = 3. *R*^2^ = displacement linearity; ${\boldsymbol{\bar{V}}}$ = mean velocity.

To analyse the behaviors of these bacteria, which resisted initial clearance, we performed single-cell trajectory analysis (SCTA) on this population (Fig. 2A and B). The position of individual bacteria was tracked between sequential frames. The majority of bacteria were unbound showing an almost constant displacement with the flow (Fig. 2C and D). Plotting their displacement over time showed a linear relationship with their average velocity exceeding 30 µm/s (Fig. 2D; Movie 2, bacteria in green circle). Despite the flow, a minority population did manage to interact with the epithelial cells, either rolling or bound. Rolling bacteria exhibited fluctuations in displacement, resulting in a less linear relationship with time (Fig. 2E and F). Their average velocity did not exceed 30 µm/s (Fig. 2F; Movie 2, bacteria in blue circle), suggesting that these bacteria established moderate binding to cells, attenuating their displacement. The term rolling was chosen to correlate to previous studies describing a similar mode of adhesion on abiotic surfaces (Thomas et al. 2004). A few bacteria managed to strongly resist displacement by the flow (Fig. 2G and H). These bacteria that exhibited stationary adhesion over time were designated as bound (Fig. 2G and J; Movie 2, bacterium in red circle). Their relationship between displacement and time was nonlinear, and their average velocity ranged from 0 to 30 µm/s. These phenotypes were not static. We observed unbound bacteria that abruptly attached to a cell (Fig. 2H) and attached bacteria that abruptly detached (Fig. 2I). To obtain a better overview of initial adhesion, we plotted mean velocity against displacement linearity (Fig. 2K). The majority of bacteria were unbound (*R*^2^ ≥ 0.95, mean velocity > 30 µm/s) with the remaining minority either rolling (*R*^2^ > 0.95, mean velocity ≤ 30 µm/s) or bound (*R*^2^ < 0.95, mean velocity ≤ 30 µm/s). This data showed that under shear stress, only a subset of bacteria successfully adhered to renal cells upon initial exposure. Adhesion could be either weak, allowing bacteria to roll on the cell surface, or strong, enabling bacteria to immobilize themselves on the cell.

### UPEC’s initial adhesion under shear stress is transient, and strengthened by the P fimbriae tip adhesin PapG

**Figure 3.**
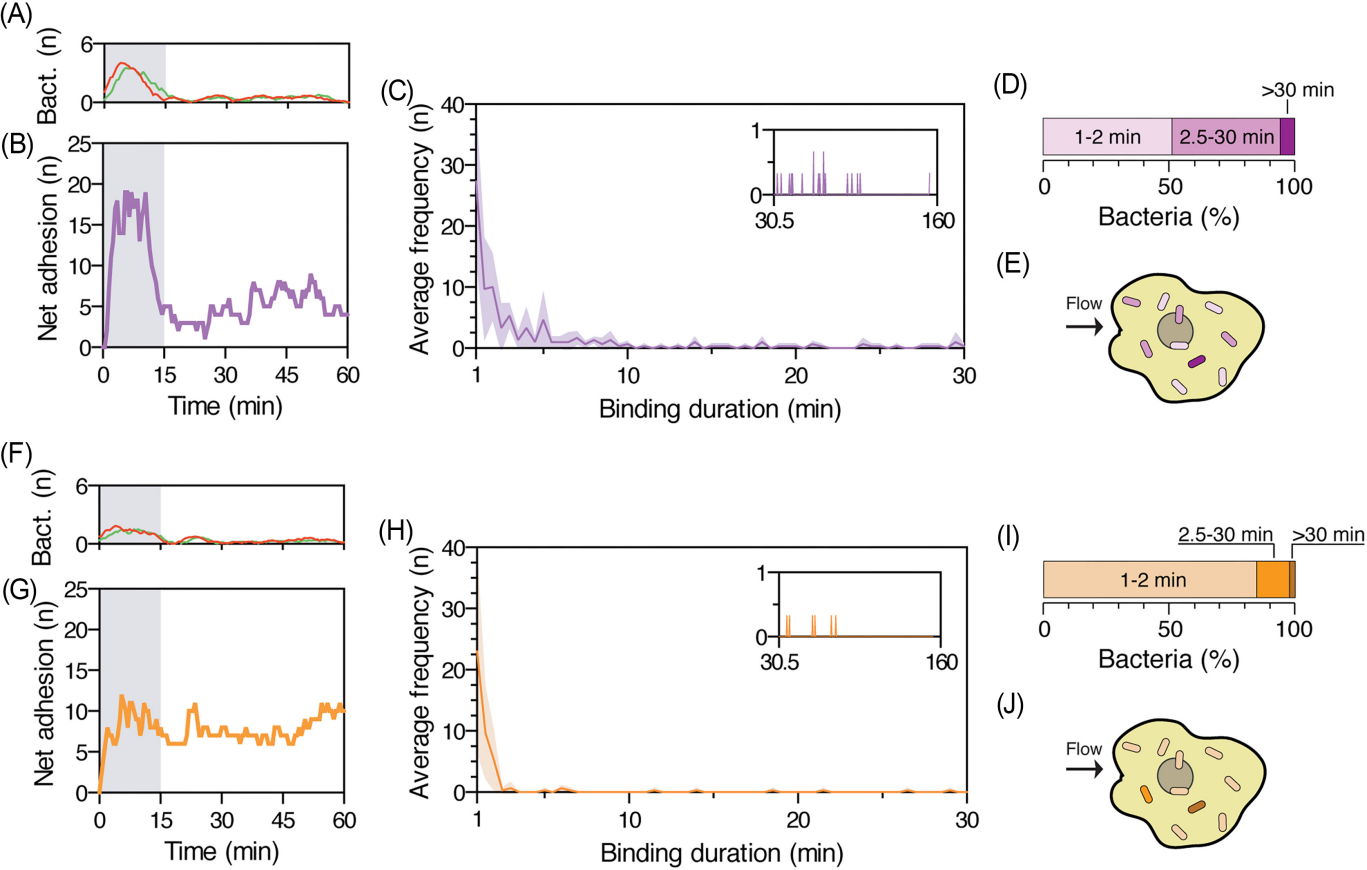
(A) Adhesion (red) and detachment (green) events per frame, and (B) net adhesion events of CFT073 wt in the PToC. Gray shade indicates the period when the inoculum flows through the microchannel. Data shown are from a 2 × 10^4^ µm^2^ area of the field of view from one representative experiment. Smoothness of the adhesion and detachment curves has been adjusted. (C) Average absolute frequency distribution of wt binding duration to renal cells between 1 and 30 min (main graph) and 30.5–160 min (inset). *n* = 3, gray shade = SD. (D) Average relative frequency of wt bacteria binding for 1–2, 2.5–30, and > 30 min to renal cells. *n* = 3. (E) Schematic representation of transient wt bacterial adhesion to renal cells. The three shades of purple in bacteria match the binding durations shown in (D). (F) Adhesion (red) and detachment (green) events per frame, and (G) net adhesion of CFT073 ∆*papG* in the PToC. Gray shade indicates the period when the inoculum flows through the microchannel. Data shown are from a 2 × 10^4^ µm^2^ area of the field of view from one representative experiment. Smoothness of the adhesion and detachment curve has been adjusted. (H) Average absolute frequency distribution of ∆*papG* binding duration to renal cells between 1 and 30 min (main graph) and 30.5–160 min (inset). *n* = 3, gray shade = SD. (I) Average relative frequency of ∆*papG* bacteria binding for 1–2, 2.5–30, and > 30 min to renal cells. *n* = 3. (J) Schematic representation of ∆*papG* transient bacterial adhesion to renal cells. The three shades of orange match the binding durations shown in (I).

With the PToC established, we wanted to understand how rolling and bound bacteria could serve as initiators for colonization. After introduction of bacteria into the PToC, it took ∼15 min for the full inoculum to flow through the microchannel. Over this period, several bacteria initiated adhesion. To quantify binding duration, we selected an area of interest (≈ 2 × 10^4^ µm^2^) and tracked for 1 h all bacteria with a duration of adhesion ≥ 1 min (Movie 3). During the 15 min inoculum infusion, we observed a high number of bacterial adhesion events, followed by numerous detachment events (Fig. 3A). After the inoculation period, adhesion and detachment events stabilized for the remaining 45 min. By calculating the total number of adherent bacteria at any given time point (net bacterial adhesion), we confirmed that a number of bacteria maintained adhesion after the inflow of the inoculum (Fig. 3B). Measuring the binding duration showed that a large proportion of bacteria managed only short-lived adhesion (Fig. 3C), 50.2 ±11.9% of bacteria adhered only for 1–2 min, while 42.8 ± 8.2% and 7 ± 4.8% adhered for 2.5–30 min and > 30 min, respectively (Fig. 3D). This data shows the huge variability in bacterial adhesion and that there is a rapid turnover of adherent bacteria. Despite these fluctuations, a small number of bacteria consolidated strong adhesion to initiate a founder population (Fig. 3E).

P fimbriae is known to be a key factor in UPEC binding to renal epithelial cells and therefore we tested the role of this adhesin in the PToC. We constructed an isogenic mutant of CFT073, which lacked the PapG tip adhesin of the P fimbriae, CFT073 ∆*papG* ∆*papG_2*, hereafter referred to as ∆*papG* (Table S1, Supporting Information). The ∆*papG* strain was inoculated into the PToC and followed for the first hour. During the inoculation phase, adhesion events were followed by an equal number of detachment events (Fig. 3F). Thereafter, net bacterial adhesion demonstrated a low but stable number of adherent bacteria during the first hour of infection even in the absence of PapG (Fig. 3G). The frequency distribution of binding duration showed, however, that most bacteria were transiently adhering for only 1–2 min (Fig. 3H). We found that 85 ± 1.6% of ∆*papG* adhered for 1–2 min, a significantly higher percentage (*P*-value = .0347) compared to wt (Fig. 3I). Only 12 ± 4.7% of the bacteria adhered for 2.5–30 min, significantly less than wt (*P-*value = .0092). Only 3 ± 3.9% of tracked ∆*papG* bacteria attached for > 30 min. Collectively, this data shows that the presence of PapG contributed to the binding duration of CFT073 in the PToC, and that bacteria unable to express PapG had a reduced ability to mediate long-term adhesion under physiological shear stress (Fig. 3J).

### Rapid proliferation and microcolony formation facilitate UPEC establishment of infection under shear stress

**Figure 4.**
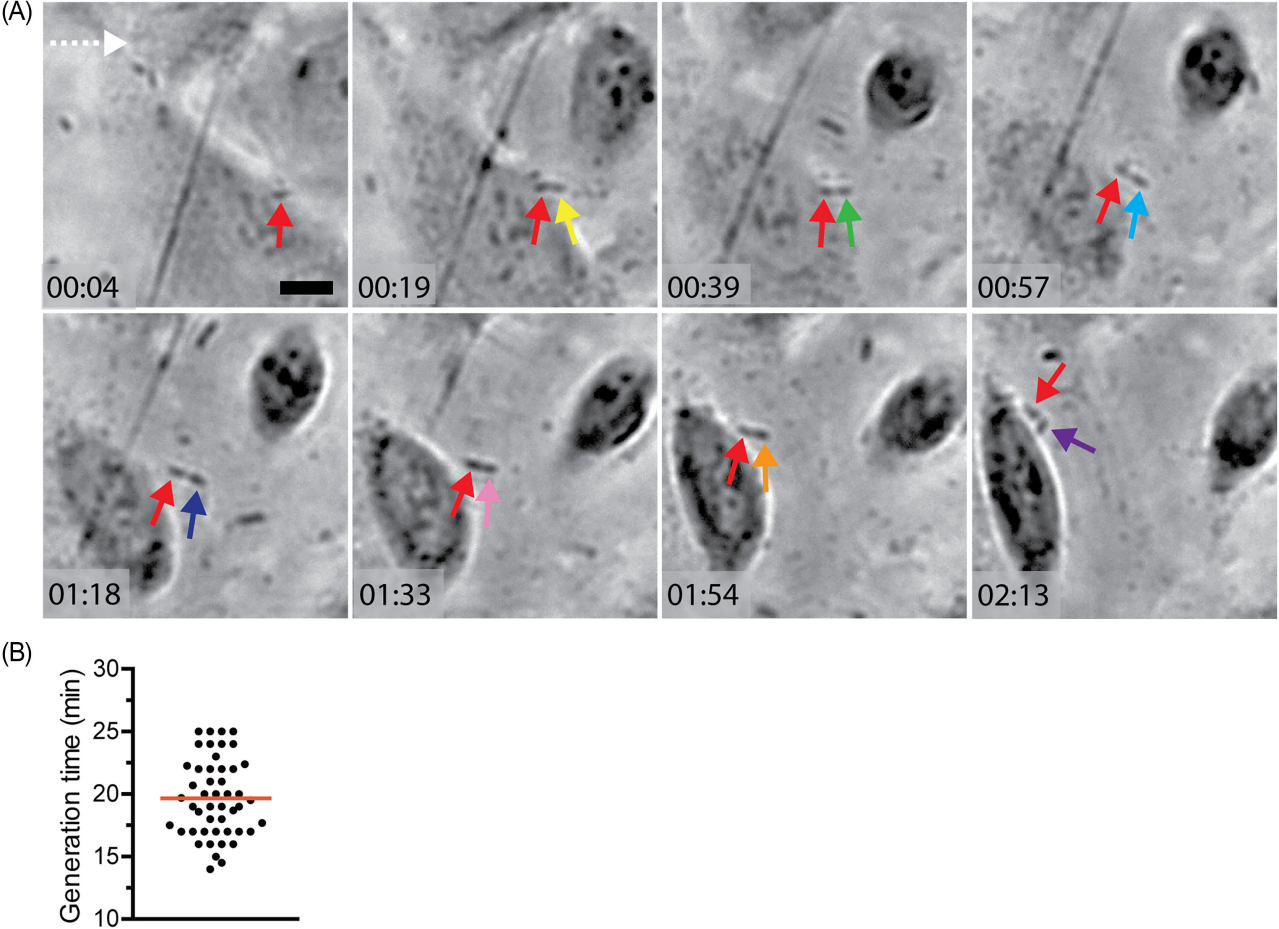
(A) A wt bacterium (red arrow) is undergoing seven sequential divisions while attached to a renal cell under flow. Arrows in other colors indicate the different daughter cells generated after each division. Selected frames from Movie 4 are shown. Flow direction (dotted white arrow) and scale bar = 10 µm apply to all figures. Time = hh:mm. (B) Generation time of wt bacteria dividing while being attached to renal cells under flow. Mean (red line) is shown. Pooled data from *n* = 3.

During the first hour of infection in the PToC, we observed a rapid turnover of adherent bacteria. Attachment and detachment events decreased after the initial inoculum had passed through the channel, but thereafter remained stable. As there was no more inflow of bacteria, this suggested that new attachment events must originate from bacterial multiplication in the channel. We analysed time-lapse recordings from CFT073 wt and observed active division in adherent bacteria (Movie 3). This is exemplified in Fig. 4(A) and Movie 4, where a bacterium attached at the initiation of infection divided seven times. After each division, one daughter cell remained adhered, while the other was released. To measure mean bacterial generation time, we tracked multiple dividing bacteria and found a mean generation time of ≈ 20 ± 3.1 min (Fig. 4B), demonstrating that adherent bacteria were rapidly dividing in the PToC. This data suggested that attached bacteria serve as seeding points, releasing newly produced daughter cells. More importantly, by dividing every ≈ 20 min, bacteria partly overcome the transient nature of their adhesion. Bacteria bound for > 20 min have sufficient time to divide and shed new bacteria in the microchannel.

**Figure 5.**
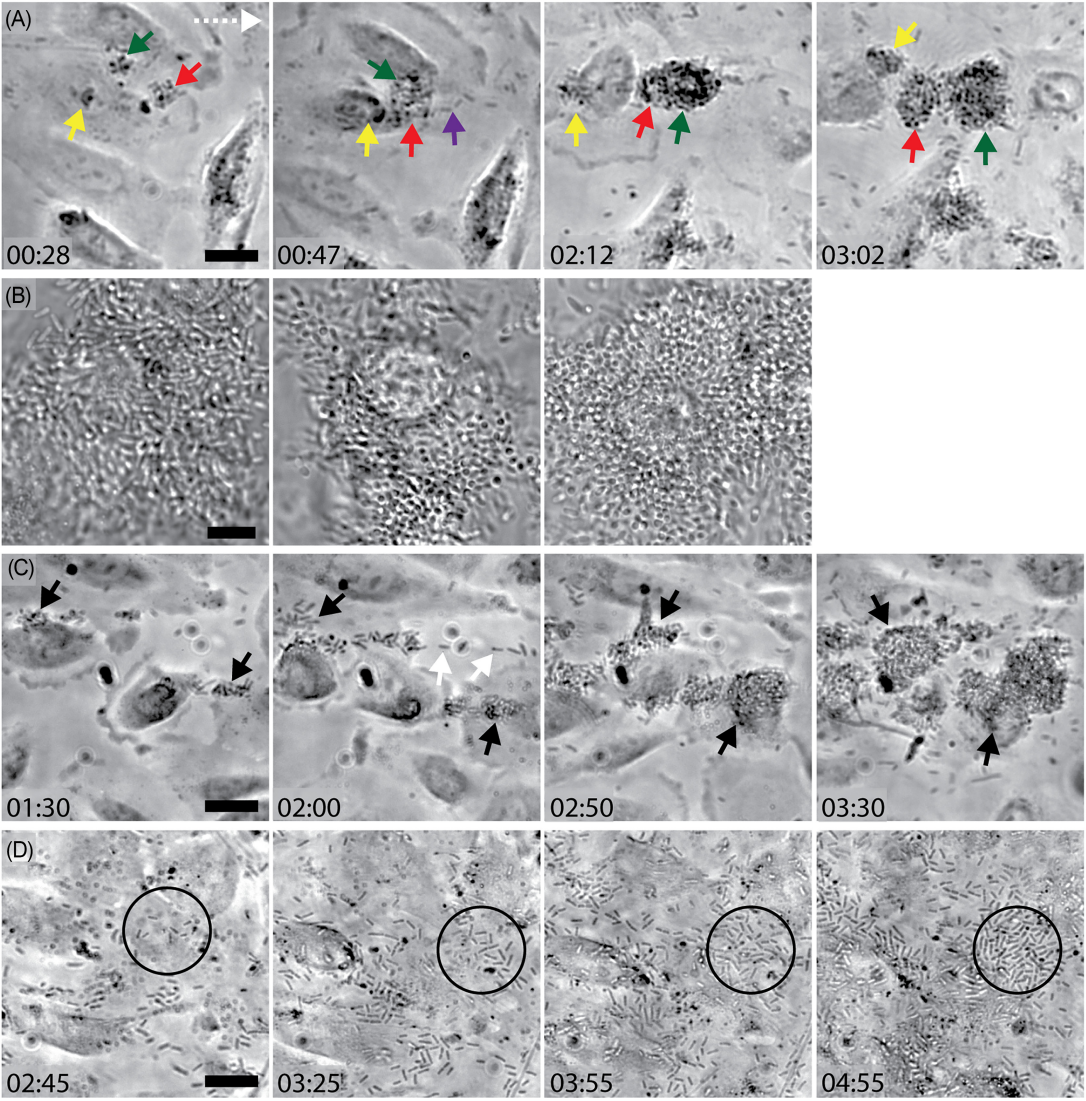
(A) CFT073 wt microcolonies (green, red, and yellow arrow) attached to a renal cell. By 47 min, two microcolonies (green and red arrow) have combined into one. Rapid proliferation in microcolonies lead to shedding of bacteria (purple arrows) in the microchannel as shown at 47 min, but it also contributed to the increase of microcolony size over time as shown at 2 h 12 min. At 3 h 2 min, the bottom part of the combined microcolony (red arrow) directly attached to a renal cell, while the upper part (green arrow) extended into the microchannel, oscillating in the flow direction. Selected frames from Movie 5 are shown. Scale bar = 20 µm, time = hh:mm. Flow direction (white dotted arrow) applies to all figures in a–d. (B) Representative CFT073 wt microcolonies at late stages of infection at 100x magnification. Scale bar = 10 µm. (C) Establishment of two CFT073 ∆*papG* microcolonies (black arrows) on renal cells under flow (1 h 30 min). Rapid proliferation lead to shedding of newly divided bacteria (white arrows) in the microchannel (2 h), and also lead to larger microcolonies (2 h 50 min). Microcolonies eventually grew to a comparable size to wt bacteria (3 h 30 min). Frames from a representative time-lapse video are shown. Scale bar = 20 µm, time = hh:mm. (D) Colonization of renal cells by ∆*fimH* under flow. Attached bacteria rapidly divided, forming a single layer of bacteria (black circle) on the cells. Scale bar a–d = 20 µm, time = hh:mm.

Occasionally, both daughter cells remained attached after division. These bacteria rapidly established prominent microcolonies within the first hour of infection (Fig. 5A; Movie 5). Time-lapse analysis showed that microcolonies expanded three-dimensionally; the bottom layer of bacteria was directly attached to the host cell, and the top layers of bacteria extending into the microchannel (Fig. 5A and Figure S1a–c, Supporting Information; Movie 5). The rapidly proliferating bacteria in microcolonies had a high shedding rate (Fig. 5A, purple arrow), with an increasing number of bacteria detected in the flow through of the PToC upon the development of microcolonies (Figure S2, Supporting Information). Unexpectedly, bacterial microcolonies exhibited prolonged binding to host cells (> 1 h), compared to single bacteria despite the shear stress (Fig. 5A and Figure S1a–c, Supporting Information). We observed several microcolonies that alternated between attachment and detachment, as they were displaced by the flow (Figure S1d, Supporting Information; Movie 5). By 3–4 h, the majority of host cells were colonized by multiple microcolonies, while other cells had only a few single bacteria attached, demonstrating a nonuniformity of colonization (Figure S3a, Supporting Information). Bacteria in mature microcolonies were oriented both horizontally and vertically to the cell surface (Fig. 5B).

Bacterial microcolonies typically remained attached to renal epithelial cells for more than an hour. As we had found that PapG enhances binding strength and prolongs single bacteria’s adhesion to renal cells, we investigated how this adhesin would affect microcolony adhesion. Time-lapse videos of infection with the ∆*papG* strain, unable to express PapG, showed that a minority of bacteria adhered to cells and proliferated with an optimal generation time of ≈ 20 min, similar to wt infection (Figure S4, Supporting Information). Daughter cells were either released in the microchannel or remained attached, eventually establishing microcolonies (Fig. 5C). Microcolonies of ∆*papG* eventually reached a size similar to microcolonies of wt bacteria (Figs 5C and 3H; 30 min). The colonization pattern was also similar to wt infection, with large microcolonies grown on some host cells and scarce colonization on others (Figure S3b, Supporting Information). Adhesion of the ∆*papG* microcolonies was significantly longer compared to the brief binding of single ∆*papG* bacteria, with microcolonies remaining attached for > 1 h. These results show that the lack of PapG did not prevent bacteria from establishing microcolonies. Microcolony formation compensated for the short-lived binding of ∆*papG* bacteria and enabled them to withstand shear stress and eventually colonize renal cells in a similar pattern to wt bacteria.

Overall, this data shows that the strong initial adhesion combined with rapid proliferation of single bacteria led to UPEC microcolony formation under shear stress. Once part of a microcolony, bacteria exhibited enhanced resistance to shear stress compared with single bacteria and firmly established colonization of renal cells. Simultaneously, these expanding microcolonies shed multiple new bacteria in the microchannel, enhancing the ability of the bacterial population to colonize.

### Interbacterial binding mediated by FimH enables UPEC to form microcolonies on renal cells under shear stress

Since P fimbriae were not shown to be critical to microcolony formation, we turned our attention to the Type 1 fimbriae, as our previous *in vivo* work had shown that the tip adhesion of Type 1 fimbriae, FimH, is involved in interbacterial binding (Melican et al. 2011). We constructed CFT073 ∆*fimH*, an isogenic mutant lacking the FimH tip adhesin, hereafter referred to as ∆*fimH* (Table S1, Supporting Information). We infused ∆*fimH* into the PToC and monitored infection with time-lapse microscopy as previously. The bacteria’s distribution of binding duration was comparable to wt and adherent bacteria proliferated with a generation time of ≈ 20 min (Figures S4 and S5, Supporting Information). Daughter cells either remained attached or were shed into the microchannel (Movie 6). Cellular colonization by ∆*fimH* lacked, however, microcolony formation but rather occurred as a single layer always directly bound to the cell surface (Fig. 5D; Movie 6). By 6.5 h, the majority of the cell surface was colonized by a bacterial monolayer (Figure S3c, Supporting Information). Occasional microcolonies could be observed in ∆*fimH* but they were rare. Complementation of the CFT073 ∆*fimH* with a constructed pBAD-*fimH* restored the ability to form microcolonies (Figure S6a and Tables S1 and S2, Supporting Information) and addition of D-mannose into the PToC, masking FimH binding, prevented CFT073 wt from forming microcolonies (Figure S6b, Supporting Information). Collectively, these data further strengthen the role of the FimH adhesin in microcolony formation. Together this indicates that FimH facilitates the formation of microcolonies and the colonization of bacteria in areas not in direct contact with the host cell surface. Although bacterial proliferation is rapid, a difficulty in forming microcolonies delays ∆*fimH* colonization, leading to a different colonization pattern.

### The role of ECM in UPEC microcolony formation

**Figure 6.**
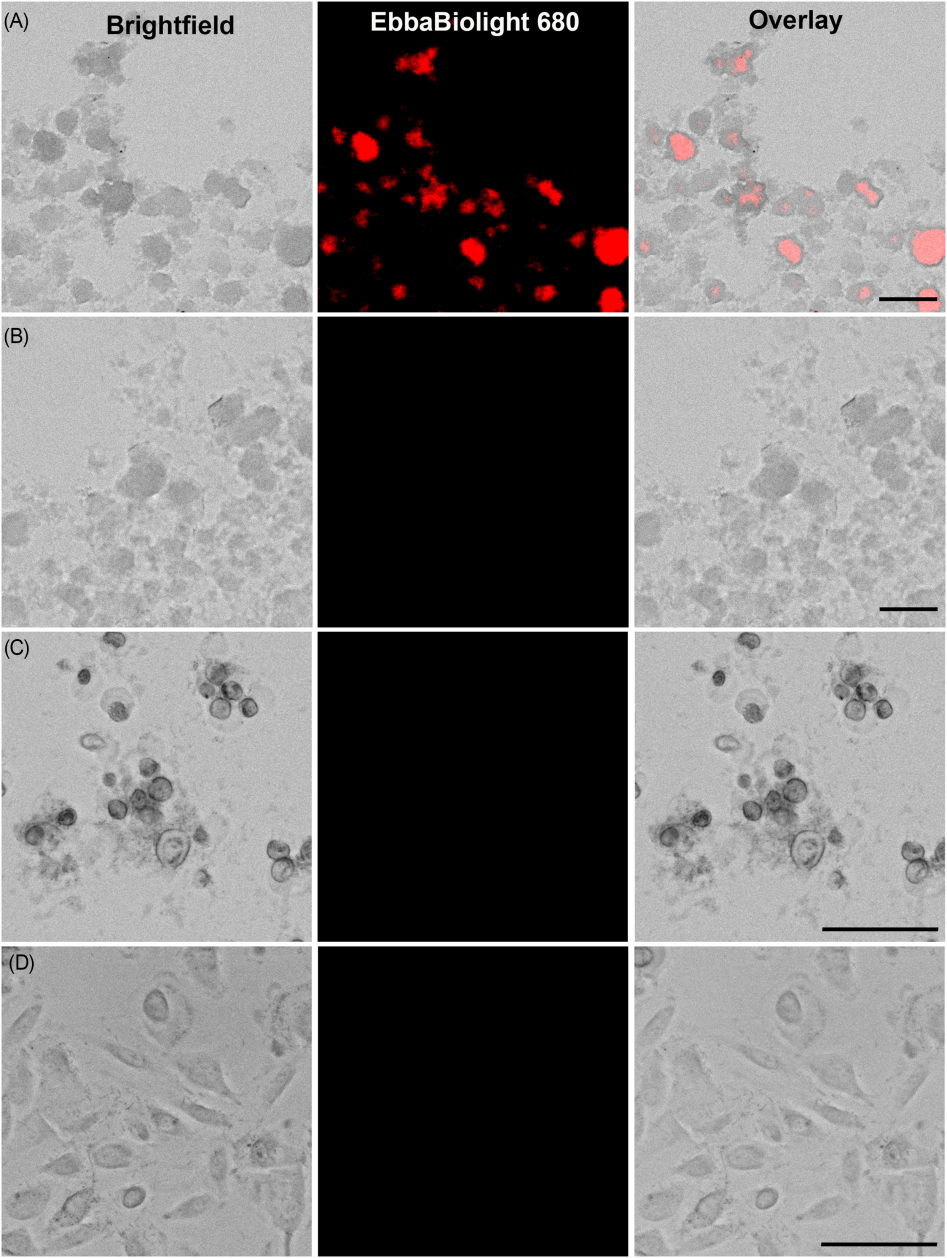
ECM detection. (A) and (B) Representative images of surface biofilm assay of CFT073 at 4 days (A) with EbbaBiolight 680 and (B) without EbbaBiolight 680 (*n* = 3). (C) and (D) Representative images of infection at 3.5 h in the PToC (C) with CFT073 and (D) CFT073 Δ*fimH* with the addition of EbbaBiolight 680 in the media (*n* = 3). Scale bars = 100 μm

Our earlier *in vivo* kidney work, and others’ work on the bladder had suggested that UPEC adopted a “biofilm-like” lifestyle *in vivo* (Justice et al. 2004, Melican et al. 2011, Conover et al. 2016, Ho et al. 2020). A biofilm lifestyle is often associated with the production of a bacterial ECM composed of biopolymers such as amyloid curli fibres (ECM-curli) and cellulose, the former with well-documented roles in promoting adhesion to biotic and abiotic surfaces (Romling et al. 1998, Bokranz et al. 2005, Flemming and Wingender 2010, DeBenedictis et al. 2016, Luna-Pineda et al. 2019). To determine if UPEC microcolonies contained ECM-curli, we used optotracing technology (Choong et al. 2016, 2021). Following manufacturers instructions, CFT073 biofilms were grown for 4 days in static culture, in the presence of EbbaBiolight 680 (Ebba680), a nontoxic, optically active fluorescent tracer molecule that reports bacterial production of ECM materials (Choong et al. 2016, 2021, Antypas et al. 2018). Visual analysis of UPEC microcolonies showed the presence of ECM-curli detected by Ebba680 (Fig. 6A and B). This indicates that UPEC microcolonies do express ECM-curli when grown in conditions favorable to biofilm formation. To determine if ECM-curli fibers were involved in the UPEC microcolony formation in the PToC in the first hours of infection, we added Ebba680 into the flow-through media of the PToC and imaged bacterial colonization over 3.5 h using fluorescence microscopy. While microcolonies formed as observed in previous experiment, no fluorescent signal indicative of ECM-curli was detected over the time course of the experiment (Fig. 6C). The same result was obtained for the ∆*fimH* strain (Fig. 6D). Collectively, this data indicates that while UPEC can form extensive microcolonies under flow, ECM-curli does not appear to play a role in the 3D microcolony formation at this very early time-frame. Together with our earlier data, this would indicate that the fitness factor enabling microcolony formation at this early stage, was Type 1 fimbriae.

## Discussion

By developing the PToC assay for UPEC colonization, we have enabeled a quantitive analysis of the role of the UPEC adhesins at the single cell level under dynamic flow. We have delineated different aspects of UPEC behavior under shear stress, including epithelial adhesion, rapid division, and microcolony formation. Adhesion of bacteria to epithelial cells is essential for infection initiation. P fimbriae-mediated adhesion prolonged binding duration to renal cells and enabled UPEC to resist shear stress for a longer time. Bacteria, which established stationary adhesion became the founders of infection. Rapid proliferation of the founder population led to the formation of microcolonies, whose structure was mediated by Type 1 fimbriae and, at least in the first hours, independent of ECM-curli expression. Rapid division in microcolonies increased the bacterial population locally and increased the shedding of bacteria, and thereby enabled a greater spread of infection. While these adhesion factors enhanced initial adhesion as well as microcolony formation, neither were found to be essential to colonization. Overall, these results demonstrated that UPEC possess a repertoire of redundant fitness and virulence determinants, which enhance bacteria’s perseverance in the dynamic environment of the proximal tubule. These *in vitro* findings strongly support our previously reported *in vivo* findings that P and Type 1 fimbriae act in synergy to mediate effective early colonization of the dynamic microenvironment of the kidney tubule (Melican et al. 2011).

With the methodological advancement of the PToC, we identified the rapid formation of UPEC microcolonies under flow. Microcolony formation has previously been shown to be critical to withstanding shear stress in blood stream infections with, e.g. *Neisseria meningitidis* (Mairey et al. 2006, Mikaty et al. 2009), but not previously implicated in UPEC kidney infection in this way. Our data shows that bacterial ECM-curli expression does not appear to be the key factor at this very early time frame, but instead demonstrates a clear role of Type 1 fimbriae and interbacterial binding in microcolony formation. Our data suggests that these microcolonies may undergo a transition from a Type 1-mediated microcolony to an ECM-curli rich microcolony over time. It suggest that we have different types of biofilms during UPEC colonization. Our results showed that FimH mediates interbacterial interactions enabling bacteria to form microcolonies and expand beyond the host cell surface. Considering that the *fim* operon is almost omnipresent in UPEC strains (Arthur et al. 1989, Rahdar et al. 2015), bacteria would rarely lack the ability to establish these early microcolonies.

The usability of organ-on-chip methodology is rapidly expanding and offers a high-resolution alternative to *in vivo* experimentation. The advantages of the model presented here are resolution, speed, and the ability to apply real-time quantative analysis. We can follow the first stages of bacterial–epithelial interaction under physiological flow and over-time, to understand the dynamics and kinetics of early colonization. Kidney infection is an ideal target for these types of approaches as shear stress is a predominant factor. There are several organ-on-chip models mimicking the liver (Sivaraman et al. 2005, Carraro et al. 2008, Novik et al. 2010), kidney (Baudoin et al. 2007, Snouber et al. 2011, Jang et al. 2013), lung (Huh et al. 2010, 2012), and intestine (Kim et al. 2012, Kim and Ingber 2013). The technology is making its way into infection research with a particular interest for vascular infections (Mairey et al. 2006, Mikaty et al. 2009, Claes et al. 2015).

Our study highlighted that UPEC utilizes multiple adhesion factors to consolidate colonization efficiently under shear stress. The PToC enabled us to monitor the dynamic interplay between host and pathogens with full spatial-temporal resolution in ways that are not feasible in traditional static cell cultures or animal models. We were able to demonstrate and quantify the transient nature of bacterial adhesion, and also investigate UPEC’s colonization strategy under shear stress. Monitoring of the infection at this level of detail is currently unattainable in animal models of kidney infection. We envision that future development of PToC with the addition of other types of host cells, both proximal and distal to the infection site, as well as different media compositions mimicking different kidney microenvironments, will help us understand the full complexity of pyelonephritis.

## Materials and methods

### Mammalian cell culture

A498 human renal carcinoma cells (ATCC^®^ HTB-44™) were cultured in RPMI 1640 medium (Sigma-Aldrich) supplemented with 10% FBS (Sigma-Aldrich) and 1% Glutamax (Gibco) and incubated at 37°C, 5% CO_2_, 95% humidity, unless stated otherwise.

### PToC setup

A slide containing six microchannels (μ-slide VI^0.4^, ibidi) was used with dimensions of 17 mm × 3.8 mm × 0.4 mm. 2 × 10^4^ A498 cells in 30 µl RPMI 1640 medium were loaded in each microchannel 1 day before an experiment and incubated for 1 h at 37°C. After microscopic confirmation of cell adhesion to the bottom of each microchannel, 60 μl medium was added in each microchannel reservoir and the slides were incubated overnight at 37°C. The following day, PToC was assembled. A 50-ml aliquot of prewarmed CO_2_-independent medium (Gibco), supplemented with 10% FBS and 1% Glutamax (CO_2_IM), was prepared. Media were additionally supplemented with 0.1% arabinose and 100 µg/ml ampicillin when strains CFT073 pBAD and CFT073 pBAD-*fimH* were tested. d-Mannose (3%) (Sigma) was added when masking FimH binding of CFT073 wt. The media aliquot was connected via a 1-mm internal diameter silicone tubing (VWR) to: (i) a tubing pump (Model ISM 80606, Ismatec), (ii) a bubble trap (IBI Scientific), (iii) an inlet (in-line luer injection port, ibidi), (iv) a microchannel of the slide seeded with cells, and (v) a collection tube. All these components and the tubing were interconnected via luer adaptors (IDEX). The pump was set at a flow rate of 75 µl/min and the flow of medium was initiated. Then, the slide was placed inside a portable 37°C incubator (ibidi) designed to fit on the stage of a phase-contrast inverted microscope (Nikon TS 100). The microscope was connected to a camera (Hamamatsu) to monitor the microchannels with time-lapse microscopy controlled by the NIS-Elements Basic Research software (version 4, Nikon).

### Flow rate calculation in PToC

The linear flow velocity of the primary renal filtrate is constant along the proximal tubule (Bonvalet and de Rouffignac 1981). Therefore, we could simulate the renal proximal tubule environment if we achieved the same linear flow velocity in the microchannel of the PToC. To calculate linear flow velocity in the proximal tubule, we used formula (i):


(1) \begin{eqnarray*} {\rm{V = Q/A}}, \end{eqnarray*}

where V = linear flow velocity, Q = flow rate, and A = cross-sectional area of the channel. To first calculate the cross-sectional area (A) of the proximal tubule, we applied formula (ii):


(2) \begin{eqnarray*} {\rm A} = \pi \times {{\rm r}^{2}}, \end{eqnarray*}

where r = radius. The inner diameter of the proximal tubule, however, decreases along this nephron segment. Thus, we can only estimate the flow rate in the first segment of the proximal tubule, which has an average internal diameter of 41.5 ± 6.2 µm in humans (Møller and Skriver 1985). Therefore, the cross-sectional area of the proximal tubule is ≈ 0.00135 mm^2^. Since the S1 segment is adjacent to the Bowman’s capsule, the flow rate of the primary filtrate in the S1 segment of the proximal tubule corresponds approximately to the Single Nephron Glomerular Filtrate Rate (SNGFR), which has been estimated between 60 and 71 nl/min in the human kidney (Oken 1982, Lazzara and Deen 2007). By inserting the SNGFR and the average cross-sectional area of the S1 proximal tubule in formula (ii), we found that the velocity of the primary filtrate in the S1 segment is ≈ 48.15 mm/min. To achieve the same velocity in a microchannel, we calculated its cross-sectional area (1.52 mm^2^), using formula (ii). The flow rate needed to maintain the same velocity in a microchannel as in the proximal tubule is approximately 73.19 mm^3^/min as calculated with formula (i) or 73.19 µl/min. Due to fine-tuning limitations of the pump, we applied a flow rate of 75 µl/min for all experiments.

According to the slide’s manufacturer, shear stress in a microchannel is calculated as


(3) \begin{eqnarray*} {\rm T}({\rm dyn/c}{\rm m}^{\rm 2}) = {\rm \eta }({\rm dyn} \times {{\rm s}/{\rm cm}^2}) \times 176.1 \times {\rm Q}({\rm ml}/{\rm min}), \end{eqnarray*}

where T = shear stress and *η* = viscosity. The viscosity of cell culture medium is ≈ 0.0079 dyn × s/cm^2^ (Rinker et al. 2001). Thus, shear stress in the microchannel is 0.104 dyn/cm^2^, which is comparable to the shear stress value in the human proximal tubule estimated in another study (Essig and Friedlander 2003).

### LIVE/DEAD viability assay

A498 cells were incubated in the PToC under 75 µl/min or static conditions in CO_2_IM for 8 h. After incubation, microchannels were disconnected from the tubing and a mix of 2.5 µM ethidium homodimer-1 (dead stain) and 0.5 µM Calcein AM (live stain) from the LIVE/DEAD™ Viability/Cytotoxicity Kit (Invitrogen) was added. Cells were incubated for 20 min at 37°C and 10 images from one microchannel per condition were captured under fluorescence microscopy, using a GFP and Cy3 filter. Live and dead cells were counted in each image and the average viability (%) was then calculated.

### Bacterial mutant and plasmid construction

Bacterial strains, plasmids, and oligonucleotides used in this study are listed in Table S1 (Supporting Information). Isogenic mutants of CFT073 were constructed with one-step inactivation of chromosomal genes (Datsenko and Wanner 2000) using the pSIM6 Red expression plasmid (Datta et al. 2006). Briefly, to construct CFT073 ∆*fimH*, the complete reading frame of *fimH* was replaced by a kanamycin resistance cassette, which was PCR-amplified from pKD4 with fimH_KOF and fimH_KOR primers. The kanamycin resistance cassette was subsequently eliminated by FLP recombination using pCP20 (Cherepanov and Wackernagel 1995). CFT073 ∆*papG* ∆*papG_2* was constructed by first replacing the complete reading frame of *papG* with a kanamycin resistance cassette, which was PCR-amplified from pKD4 with papG_KOF and papG_KOR primers. Then, the complete reading frame of *papG_2* was replaced by a chloramphenicol resistance cassette that was PCR-amplified from pKD3 with papG_KOF and papG_KOR primers. Both kanamycin and chloramphenicol cassettes were subsequently eliminated with FLP recombination. All deletions were confirmed by PCR, using fimH_F, fimH_R, papG_F, papG_R, papG2_F, papG2_R primers, and by sequencing. PCR reactions to amplify antibiotic resistance cassettes were performed with Phusion DNA polymerase (NEB) at 55°C annealing temperature. PCR confirmation reactions were performed using illustra Taq PCR beads (GE Healthcare) at 58°C annealing temperature. To construct complementation vector pBAD-*fimH*, the *fimH* ORF was cloned into pBAD under the control of an arabinose-inducible promoter. Briefly, *fimH* ORF was PCR-amplified with SacI_fimH_FW and HindIII_fimH_RV primers (Table S1, Supporting Information) using gDNA from CFT073 as a template and ligated into pBAD that was previously linearized by double digestion with SacI and HindIII restriction enzymes (NEB). Electrocompetent CFT073 Δ*fimH* were transformed with pBAD and pBAD-*fimH*. Construction of pBAD-*fimH* was confirmed by sequencing. Control experiments were performed with an empty pBAD vector.

### Bacterial growth curves

Growth curves were prepared by diluting LB overnight cultures 1:100 in fresh LB, pelleting bacteria at 8000 rpm for 5 min, and resuspending in either CO_2_IM or LB. A volume of 150 µl of each resuspension was transferred in a 96-well plate in triplicates, incubated in a plate reader (Biotek) at 37°C, measuring OD_600_ every 10 min for 5 h.

### Testing fimbrial expression in bacterial mutants

Exponential phase bacterial cultures in CO_2_IM were pelleted and concentrated 100 times in saline or saline + 0.05 M methyl-mannopyranoside (Sigma). For yeast agglutination, 25 µl of the saline or saline + 0.05 M methyl-mannopyranoside resuspension were transferred on a microscopy glass slide and mixed with either an equal volume of fresh baker’s yeast suspension in saline (15 mg/ml) or saline only (negative control) for 30 s. For blood agglutination, 25 µl of the saline resuspension was transferred on a microscopy slide and mixed with an equal volume of a 2% suspension of isolated human red blood cells in saline or with saline only (negative control). Results for both yeast and blood agglutination were observed macro- and microscopically.

### Infection in PToC

One microchannel seeded with A498 cells was used per condition tested. Each microchannel was exposed to 75 µl/min of CO_2_IM as described above. To prepare the bacterial inoculum an LB overnight culture was diluted 1:100 in 10 ml prewarmed CO_2_IM and incubated at 37°C under shaking conditions to an OD_600nm_ ≈ 0.4–0.5. Then, 0.5 ml (≈ 2 × 10^8^ cfu) of the exponential phase culture was slowly introduced over the course of 1 min in the microchannel with a syringe via the inlet (*t* = 0 of the infection). This corresponds to a multiplicity of infection (MOI) of 10 000:1 (bacteria: renal cells). For a detailed description on appropriate MOI selection, see Supplementary Note 2. A random microscopic field of view was chosen along the central lane of the microchannel and infection was then monitored with time-lapse phase-contrast microscopy at 40x magnification. Alternatively, infection was monitored by manually capturing images at selected time points. Samples were collected at the desired time points by placing a 1.5-ml microcentrifuge at the outlet and collecting medium exiting the microchannel for 2 min (≈ 175 µl). Collected samples were immediately transferred to a 96-well plate, and OD_600_ was measured in a plate reader (TECAN).

### Time-lapse microscopic analysis of bacterial colonization

All time-lapse microscopy videos were recorded from three biological replicates per strain at either 10 frames per second (fps), or 1 or 2 frames per minute (fpm). Time-lapse videos were analyzed in ImageJ v1.46r (Schneider et al. 2012). To perform SCTA of bacteria entering the microchannel, 2 × 10 s time-lapse videos per biological replicate were analyzed. A starting area (78.98 µm × 209.93 µm) was selected on the leftmost part of the field of view (Fig. 2B). Using the ImageJ plugin MTrackJ (Meijering et al. 2012), all single bacteria found within this area on the first frame (*t* = 0 s) were followed either until the last frame of the video (*t* = 10 s) or until they exited the field of view. Based on bacteria’s trajectories, their displacement per 0.1 s and mean velocity ($\bar{V}$) were calculated. *R*^2^ of displacement vs. time was calculated to assess linearity.

To measure the duration of bacterial adhesion to renal cells, we randomly selected two areas of 1 × 10^4^ µm^2^ each, in the microscopic field of view. All bacteria that landed on renal cells in this area between 0 and 60 min of the infection were tracked using MTrackJ. Out of all tracked bacteria, we further analyzed only the ones attaching for at least ≥ 1 min. Adhesion events for each frame were calculated based on the number of new bacteria found attached and detachment events based on the number of bacteria detaching per frame. The smoothness of the attachment and detachment curve was adjusted with the method of Savitzky and Golay (1964) to reduce noise and improve understanding of the graphs. Net adhesion corresponds to the total number of bacteria found attached per frame. Binding duration for each bacterium was calculated based on the number of frames that it remained attached to a cell. The absolute frequency was calculated with the center of the first bin at 1 min, and a bin width = 0.25. Relative frequency for binding duration was calculated by arbitrarily categorizing bacteria in 3 binding duration ranges: (i) 1–2 min, (ii) 2.5–30 min, and (iii) > 30 min. Based on the total number of bacteria attaching for at least ≥ 1 min, the % of bacteria in each binding duration range was calculated.

To measure the generation time of dividing bacteria, time-lapse recordings at 1 or 2 fpm were analyzed to identify bacteria that remained attached in our field of view for at least two sequential divisions. The elapsed time between the two frames that the two sequential divisions occurred was defined as the generation time.

For microcolony analysis, the full microscopic field of view (473 µm × 473 µm) from time-lapse videos at 1 or 2 fpm was examined. After representative microcolonies were selected, the field of view was cropped around them for clarity.

The rounding onset of individual host cells was determined using MTrackJ on time-lapse videos. We calculated % healthy cells by dividing the number of cells with healthy morphology at each time point with the total number of cells in the field of view. Healthy cell curves (%) were constructed for three biological replicates per strain. The average curve was then calculated for each strain.

### Surface biofilm assay and ECM staining by EbbaBiolight

The surface biofilm assay was performed as described by the manufacturer (https://www.ebbabiotech.com/collections/ebbabiolight). Briefly, cover glasses (Menzel Gläser) were sterilized with 70% ethanol and modified to fit inclined in 12 well plates (Corning). Overnight culture of CFT073 was diluted 1:100 in fresh LB without salt medium in the 12 well plates with the inclined coverslips. EbbaBiolight 680 (Ebba Biotech, Sweden) (0.5 μl/ml) was added to experimental wells but not control wells. Plates were incubated for 4 days at 37°C. After 4 days, the coverslips were washed twice in PBS and fixed with 4% PFA for 1 h at room temperature. The coverslips were washed twice in PBS, then one side of the coverslip was cleaned with 70% ethanol drenched wipes (Kimtech) before the other side was mounted against microscope slides (VWR) with mounting medium (Dako). The slides were imaged in Lionheart™ FX Automated Microscope (Ramcon, Sweden) with fluorescence from EbbaBiolight 680 detected using the propidium iodide channel (Excitation 531 nm, Emission 647 nm).

### ECM detection in PToC

PToC was assembled as described above, with 0.5 μl/ml EbbaBiolight680 (Ebba Biotech) added to the flow-through medium. The Lionheart FX microscope (BioTek) was used to monitor the infection. Montage images of the middle section of the PToC were taken at infection time 0.5, 1.5, 2.5, and 3.5 h with brightfield and propidium iodide channel (excitation 531 nm, emission 647 nm).

### Movie editing

Representative frames or full-length time-lapse recordings of the full microscopic field of view (473 µm × 473 µm) were exported as 16-bit Tiff files using NIS-Elements software. Using ImageJ, Tiff files were imported as a stack, cropped around the infection area of interest, and trimmed in length. Brightness and contrast were adjusted linearly. Bacteria and microcolonies were annotated with circles and an ID number using MTrackJ.

### Statistics

All statistical analysis was performed using Prism 6. The mean relative frequencies of binding duration between strains were compared with *t*-test and healthy cells (%) curves were compared with the log-rank test.

## Authors’ contributions

H.A. and A.R.D. conceived and designed the study. H.A. performed all experiments for Figs 1–5. K.M. and T.Z. designed experiments for Fig. 6. T.Z. performed the experiments for Fig. 6 with support from F.X.C. H.A., T.Z., F.X.C., K.M., and A.R.D. analyzed the data. H.A., K.M., and A.R.D. wrote the manuscript with input from T.Z. and F.X.C.

## Supplementary Material

xtad007_Supplemental_Files
