# Supplementary material for: Dynamic single cell analysis in a proximal-tubule-on-chip reveals heterogeneous epithelial colonization strategies of uropathogenic Escherichia coli under shear stress
Source: FEMS Microbes. 2023 Mar 3;4:xtad007. doi: 10.1093/femsmc/xtad007 (PMC10117878; doi:10.1093/femsmc/xtad007)
Supplement: xtad007_Supplemental_Files [file xtad007_supplemental_files.zip › 230220_Antypas_et_al_Supplementary.docx]

**Supplementary Information**

**Dynamic single cell analysis in a proximal-tubule-on-chip reveals heterogeneous epithelial colonization strategies of uropathogenic *Escherichia coli* under shear stress**

Haris Antypas^1,2,3^, Tianqi Zhang^1,2^, Ferdinand X. Choong^1,2^, Keira Melican^1,2,*^ and Agneta Richter-Dahlfors^1,2,*^

**^1^** AIMES - Center for the Advancement of Integrated Medical and Engineering Sciences, Karolinska Institutet and KTH Royal Institute of Technology, Stockholm, Sweden

**^2^** Department of Neuroscience, Karolinska Institutet, Stockholm, Sweden

**^3^** Current address: SCELSE-Singapore Centre for Environmental Life Sciences Engineering, School of Biological Sciences, Nanyang Technological University, Singapore

**Table of contents Page**

Supplementary Figure 1 2

Supplementary Figure 2 3

Supplementary Figure 3 4

Supplementary Figure 4 5

Supplementary Figure 5 5

Supplementary Figure 6 5

Table S1 6

Table S2 7

Supplementary References 7

Movie legends 8

**
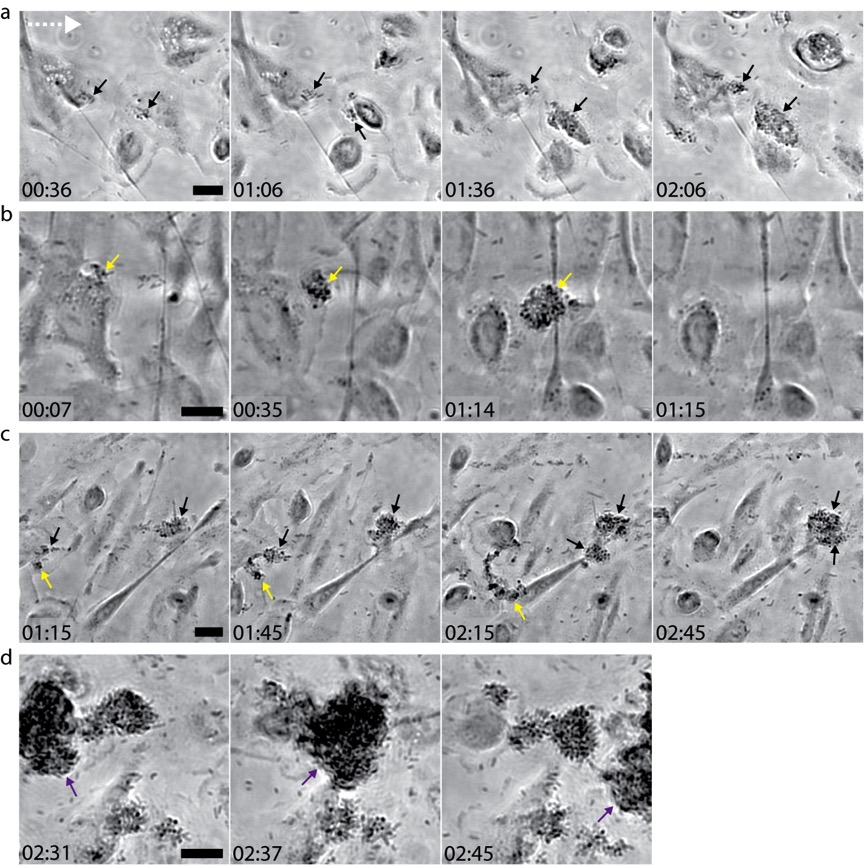
**

**Fig. S1. (a-c)** Establishment and development of CFT073 wt microcolonies on renal cells under flow. Black arrows indicate colonies that withstood shear stress throughout the time-lapse recording and yellow arrows indicate colonies that were displaced eventually outside our field of view. Representative frames were selected from time-lapse videos from 3 biological replicates. **(d)** A bacterial colony (purple arrow) alternating between attachment and detachment, as it is gradually displaced by the flow. Selected frames from Movie 5 are shown. Flow direction (white arrow) applies to all figures. Scale bars =20 µm, time = hh:mm.

**Figure S2.** Quantification of CFT073 wt shed from adherent bacteria in PToC by measuring the OD_600nm_ of samples exiting the microchannel. Blank is shown as a red dashed line for comparison. n = 1

**
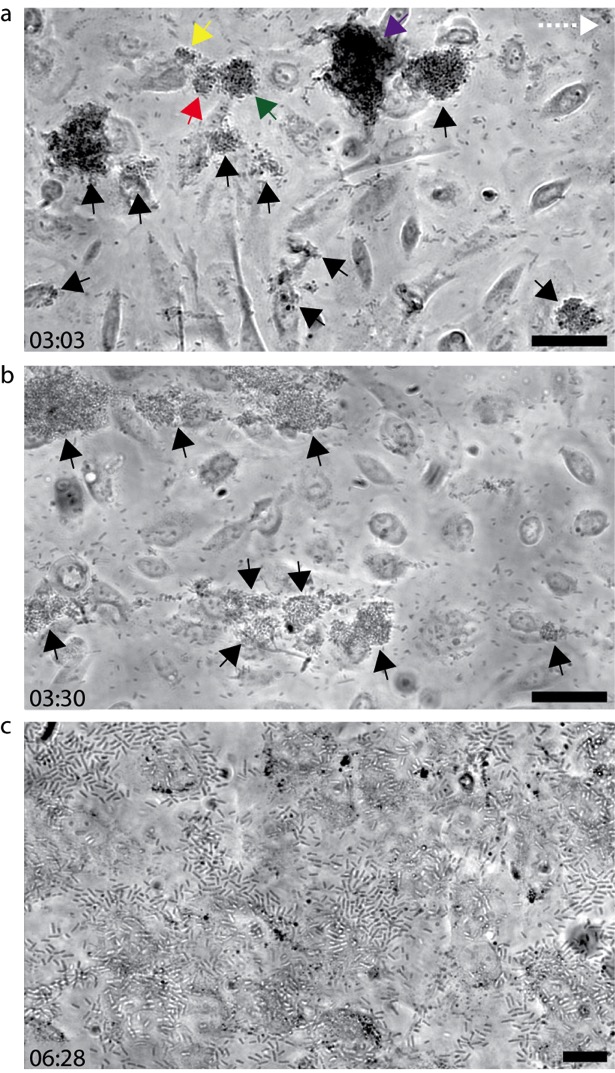
**

**Fig. S3. (a)** Overview of CFT073 wt colonization on renal cells. All arrows, regardless of colour, indicate bacterial colonies. Arrows in colour correspond to the colonies annotated with the same colour in Movie 4. Scale bar = 50 µm, time = hh:mm. Flow direction (white dotted arrow) applies to all figures. **(b)** Overview of CFT073 ∆*papG* colonization on renal cells. Bacterial colonies are annotated with black arrows. Scale bar = 50 µm, time = hh:mm **(c)** Overview of CFT073 ∆*fimH* infection, where bacteria colonize almost the entire cell surface without forming dense, 3-dimensional colonies. Scale bar = 20 µm, time = hh:mm.

**Fig. S4.** Generation time of CFT073 ∆*papG* and ∆*fimH* attached to renal cells under flow conditions. Pooled data from n = 3 per strain is shown. Red line = mean

**Fig. S5.** Average absolute frequency distribution of ∆*fimH*'s binding duration to renal cells.

n = 3, grey shade = SD.


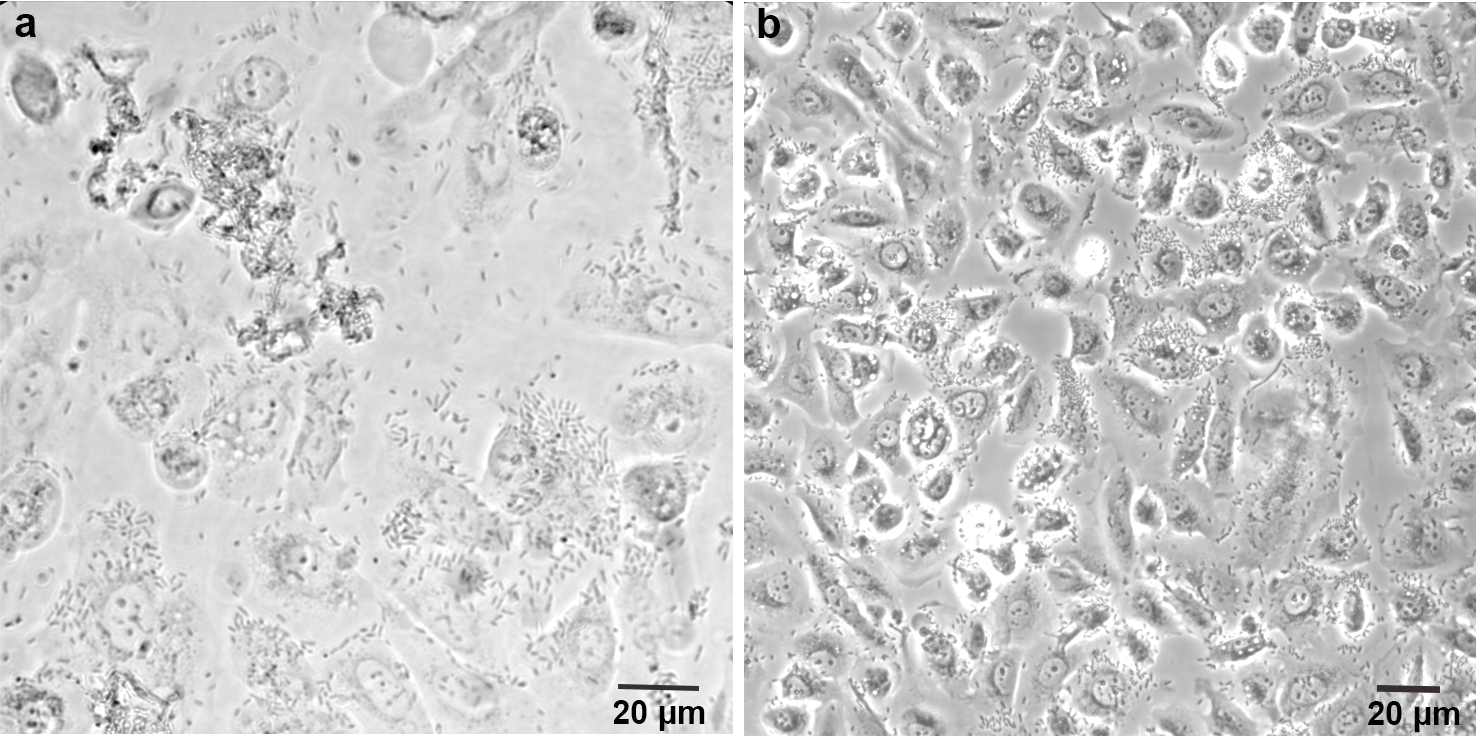


**Fig. S6.** Representative images of colonization of A498 renal epithelial cells in PToC with **a**) CFT073 Δ*fimH* pBAD-*fimH* complemented mutant induced with 0.1% arabinose at 5 h of infection (n=4) and **b**) CFT073 wt + 3% D-mannose at 6.5 h (n=3)

| **Table S1. Bacterial strains, plasmids and oligonucleotides used in this study** | | | | | |  |  |  |
| --- | --- | --- | --- | --- | --- | --- | --- | --- |
| **Strains** | | **Genotype** | | | | **Reference** | | |
| CFT073 (wt) | | Pyelonephritic *Escherichia coli* clinical isolate (ATCC® BAA-2503™) | | | | *(1)* | | |
| Δ*fimH* | | CFT073 Δ*fimH* | | | | This work | | |
| Δ*papG* | | CFT073 *ΔpapG* Δ*papG_2* | | | | This work | | |
| Δ*fimH* pBAD | | CFT073 Δ*fimH* pBAD | | | | This work | | |
| Δ*fimH* pBAD-*fimH* | | CFT073 Δ*fimH* pBAD-*fimH* | | | | This work | | |
| **Plasmid** | | **Antibiotic marker** | | | | | | |
| pSIM6 | | Amp^R^ | | | | | | *(2)* |
| pKD4 | | Amp^R^, Kan^R^ | | | | | | *(3)* |
| pKD3 | | Amp^R^, Cm^R^ | | | | | | *(3)* |
| pCP20 | | Amp^R^, Cm^R^ | | | | | | *(4)* |
| pBAD/MYc-His | | Amp^R^ | | | | | | Thermo Scientific |
| pBAD-*fimH* | | Amp^R^ | | | | | | This work |
| **Oligos** | | **Sequence (5'-3')** | | | | | | |
| fimH_KOF | | GTGATTAGCATCACCTATACCTACAGCTGAACCCAAAGAGGTGTAGGCTGGAGCTGCTTC | | | | This work | | |
| fimH_KOR | | TAATATTGCGTACCTGCATTAGCAATGCCCTGTGATTTCTATGGGAATTAGCCATGGTCC | | | | This work | | |
| papG_KOF | | ATACCTAAATGAATAACTGTAATTACGGAAGTGATTTCTGGTGTAGGCTGGAGCTGCTTC | | | | This work | | |
| papG_KOR | | CAGATATCCACAACACTCTATCCGGCTCCGGATAAACCATATGGGAATTAGCCATGGTCC | | | | This work | | |
| fimH_F | | CTATACCTACAGCTGAACCC | | | | This work | | |
| fimH_R | | TATTGCGTACCTGCATTAG | | | | This work | | |
| papG_F | | TGCTATCCATCCTTTTTACA | | | | This work | | |
| papG2_F | | GAGCATCAATGGTCTTTACA | | | | This work | | |
| papG_R | | GGTAACAAAAACCATAAGCA | | | | This work | | |
| SacI_fimH_FW | | ATAGAGCTCATGATTGTAATGAAACGAG | | | | This work | | |
| HindIII_fimH_RV | | ATAAAGCTTTTATTGATAAACAAAAGTCAC | | | | This work | | |
|  | |  | | | |  | | |
|  | |  | | | |  | | |
| **Table S2. Yeast and blood agglutination of UPEC CFT073** | | | | | |  |  |  |
|  | **Yeast** | | **Yeast + MMP^1^** | | **RBC^2^** |  |  |  |
| **CFT073 (wt)** | + | | − | | + |  |  |  |
| **Δ*fimH*** | − | | − | | + |  |  |  |
| **Δ*fimH* pBAD** | - | | - | | + |  |  |  |
| **Δ*fimH* pBAD-*fimH*** | + | | - | | + |  |  |  |
| **Δ*papG*** | + | | − | | − |  |  |  |
| ^1^ Methyl-mannopyranoside | | | |  | |  |  |  |
| ^2^ Human red blood cells | | | |  | |  |  |  |

**Supplementary References**

1. H. L. T. Mobley, D. M. Green, A. L. Trifillis, D. E. Johnson, G. R. Chippendale, C. V. Lockatell, B. D. Jones, J. W. Warren, Pyelonephritogenic Escherichia coli and Killing of Cultured Human Renal Proximal Tubular Epithelial Cells : Role of Hemolysin in Some Strains, *Infect. Immun.* **58**, 1281–1289 (1990).

2. S. Datta, N. Costantino, D. L. Court, A set of recombineering plasmids for gram-negative bacteria, *Gene* **379**, 109–115 (2006).

3. K. A. Datsenko, B. L. Wanner, One-step inactivation of chromosomal genes in Escherichia coli K-12 using PCR products., *Proc. Natl. Acad. Sci. U. S. A.* **97**, 6640–6645 (2000).

4. P. P. Cherepanov, W. Wackernagel, Gene disruption in Escherichia coli: TcR and KmR cassettes with the option of Flp-catalyzed excision of the antibiotic-resistance determinant, *Gene* **158**, 9–14 (1995).

**MOVIE LEGENDS**

**Movie 1.** CFT073 wt bacteria entering a PToC microchannel. A stream of rapidly expelled bacteria, as well as bacteria expelled at lower velocity close to the cell surface, are shown. A representative, full-length 10-s time-lapse video from n = 1 is shown. Recording and playback speed = 10 fps, scale bar = 50 µm, white arrow = flow direction.

**Movie 2.** Tracking of single CFT073 wt bacteria shown in movie 1 to identify modes of adhesion to renal cells. Bound (red circle), rolling (blue circles), and unbound bacteria (green circles) are shown. Displacement graphs in Fig. 2b, d & h derived from bacteria 2:1, 3:1 and 4:1 respectively, shown in this movie. Recording = 10 fps and playback speed = 7 fps, scale bar = 50 µm, white arrow = flow direction.

**Movie 3.** Representative clip from a time-lapse recording of CFT073 wt bacteria during the 1^st^ hour of the infection. Bacteria transiently attaching (black circle), 2 bacteria dividing while bound (red circle) and their daughter cells (blue circle) are shown. Recording speed = 1 fpm, playback speed = 10 fps, Scale bar = 30 µm, white arrow = flow direction, time = hh:mm.

**Movie 4.** Representative clip from a time-lapse recording of a CFT073 wt bacterium (red circle) rapidly proliferating 7 times under flow in the course of 2 h 23 min, while being attached to a renal cell. After each division, one of the 2 daughter cells is highlighted with a circle of a different colour. Recording speed = 2 fpm, playback speed = 10 fps, scale bar = 10 µm, white arrow = flow direction, time = hh:mm.

**Movie 5.** Representative clip from a time-lapse recording of CFT073 wt bacteria forming 3 microcolonies (green, red, yellow circles) while being attached on a renal cell. A 4^th^ colony (purple circle) with weaker binding to cells crosses our field of view in the flow direction. Circle colours correspond to the arrow colours shown in Fig. 4 and Fig. S5a. Recording speed = 2 fpm, playback speed = 10 fps, scale bar = 15 µm, white arrow = flow direction, time = hh:mm.

**Movie 6.** Representative time-lapse recording of CFT073 ∆*fimH* bacteria colonizing renal cells. Recording speed = 2 fpm, playback speed = 10 fps. Scale bar = 15 µm, white arrow = flow direction, time = hh:mm.
