# Supplementary material for: Dynamic single cell analysis in a proximal-tubule-on-chip reveals heterogeneous epithelial colonization strategies of uropathogenic Escherichia coli under shear stress
Source: FEMS Microbes. 2023 Mar 3;4:xtad007. doi: 10.1093/femsmc/xtad007 (PMC10117878; doi:10.1093/femsmc/xtad007)
Supplement: xtad007_Supplemental_Files [file xtad007_supplemental_files.zip › FEMSMC-2022-027_reviews_and_author_response.docx]

**Reviews of manuscript FEMSMC-2022-027**

**Reviewer 1**

**Recommendation: Minor revision**

The authors present a novel method of investigating the interaction between mammalian and bacterial cells in a controlled environment. The method seems to have been well thought out to replicate the target environment as well as can be done in an in-vitro system. I only have a couple of minor comments to a solid manuscript.

- Given the short time frames of the experiment, I was curious if the authors considered varied media composition after the cell layer was stabilised to further mimic the target environment? The bacterial cells will likely react differently to even small nutrient changes, especially in the generation/maintenance of microcolonies.

- Check the usage of shear/sheer throughout the manuscript. There are a few occurances of the incorrect term.

- Line 386 Utilises, not utalises

**Reviewer 2**

**Recommendation: Major Revision**

To study the contribution of P- and type 1 fimbriae to initial UPEC-host interaction under physiologically relevant conditions, the authors employed a proximal-tubule-on-chip (PToC) based on the A-498 human renal epithelial cell line. Shear stress generated by the primary filtrate flow in the proximal tubule was mimicked and adherence of infusing UPEC under these conditions was studied by time-lapse microscopy using model isolate CFT073. The majority of UPEC did not interact with A-498 cells was rapidly cleared. Only a small number of bacteria resisted the flow and was slowly moving in the flow either rolling or weakly attaching to the epithelial cells. Only a few bacteria strongly interacted under shear stress with the epithelial cells.The interaction phenotype of UPEC with A-498 cells was reversible: bound cells can detach again and vice versa. Initially adherent bacteria displayed great fluctuation regarding adherence with a rapid turnover of adherent bacteria. Only a small number of UPEC cells maintained to be strongly attached to the kidney cells and initiated a founder population. Attached bacteria were rapidly dividing and if they attached to A-498 cells for more than 20 min have sufficient time to divide and either shed new bacteria or to expanded three-dimensionally. Such bacterial microcolonies were, despite the shear stress, attached to host cells for longer periods of time (more than one hour) compared to single bacterial cells.

Repetition of these analyses with an isogenic papG double mutant showed that the presence of the P fimbrial adhesin contributes to the duration of bacterial binding to A-498 cells and that bacteria lacking the P fimbrial adhesin were inferior in long-term adhesion under shear stress. The papG deletion mutant was also able to establish microcolonies. Though the duration of bacterial initial binding was shorter, microcolony formation

compensated for that and promoted colonization of the kidney cell line in a similar pattern to the wild type.

Deletion of fimH coding for the type 1 fimbrial adhesin did not affect the range of binding duration of single cells or bacteria multiplication. However, cellular colonization by ΔfimH did not involved microcolony formation and rather occurred as a bacterial monolayer directly attaching the epithelial cell surface.

Optotracing of microcolonies of the different mutants tested in this study in the presence of EbbaBiolight 680 did not allow to detect extracellular matrix production in the microcolonies suggesting that extracellular matrix production does not contribute to early microcolony formation stages under these conditions.

This study reports on the synergistic effects that type 1- and P fimbrial expression has during the early steps of urothelial colonization, which is interesting. The idea to use an experimental system that allows to test the effect of shear stress is sensible and should be a good tool to mimick relevant conditions in the proximal tubule.

1. As a control, a fimHpapG1papG2 triple deletion mutant needs to be tested in the PToC system.If the authors are right, the triple deletion mutant would be a negative control.

2. It is interesting that deletion of the type 1 fimbrial adhesin gene affects interbacterial recognition thus promoting microcolony formation. It is known that type 1 fimbria expression contributes to pellicle formation in static cultures, but this reviewer thought that such type 1 fimbria-mediated interactions were due to interactions between the fimbrial filaments (and not due to the action of the fimbrial adhesin). Against this background, a higher resolution analysis of the cell surface exposed structures will be interesting to get an idea which structures may be involved in interbacterial interaction. Are fimbrial filaments produced at all in the mutants and are they involved in this type of interaction?

3. If indeed, FimH-mediated binding to mannosyl residues-containing structures plays a role here, microcolony formation of type 1 fimbriae-expression bacterial should be reduced in the presence of D-Mannose in the interaction medium. This should be tested.

4. Molecular biology studies always benefit when analysing deletion mutants from the fact that these mutations can be complemented to prove the relevance and specificity of the mutation. It would therefore also be useful and desirable in this study if appropriate complemented mutants were included.

5. The authors tested the interaction of UPEC strain CFT073 with non-differentiated A-498 epithelical cells. Proximal tubular cells are the most abundant cell type of all renal tubule epithelial cells. Nevertheless, there are also less frequently present cell types, incl. TAL and DCT cells as well as AQP2-expressing cells (CNT, PC, and IMCD) that can be found in the renal epithelium. Some of them have been suggested to be important for interacting with invading UPEC (e.g. intercalating cells). Similarly, there is also shear stress acting on UPEC when they colonized the bladder. Did the authors try to test bladder epithelial cells lines in the PToC system as well or to mix A-498 cells with other renal epithelial cells lines to see whether certain cell types are more relevant to interacting with UPEC than others?

**Author's Response to Decision Letter for (FEMSMC-2022-027)**

Reviewer 1

Comments to the Author

The authors present a novel method of investigating the interaction between mammalian and bacterial cells in a controlled environment. The method seems to have been well thought out to replicate the target environment as well as can be done in an in-vitro system. I only have a couple of minor comments to a solid manuscript.

Comment 1 Given the short time frames of the experiment, I was curious if the authors considered varied media composition after the cell layer was stabilised to further mimic the target environment? The bacterial cells will likely react differently to even small nutrient changes, especially in the generation/maintenance of microcolonies.

Response: We agree that nutrient composition is a very relevant factor in the infectious kidney environment. In the very short time-frame of this set of experiments, we have not altered the media composition. However, we are looking to follow-up on the use of different media as well as the presence of different cell types in subsequent projects. To emphasize the usefulness of the PToC for such studies, we have altered the text as follows:

Changes made to the manuscript

Discussion, p 16, lines 406-407: ”We envision that future development of PToC with the addition of other types of host cells, both proximal and distal to the infection site, as well as different media compositions mimicking different kidney microenvironments, will help us understand the full complexity of pyelonephritis.”

Comment 2 Check the usage of shear/sheer throughout the manuscript. There are a few occurances of the incorrect term.

Response: This has now been corrected, see lines 40, 47, 58, 130

Comment 3 Line 386 Utilises, not utalises

Response: Thank you, corrected (now line 398).

Reviewer 2

Comments to the Author

To study the contribution of P- and type 1 fimbriae to initial UPEC-host interaction under physiologically relevant conditions, the authors employed a proximal-tubule-on-chip (PToC) based on the A-498 human renal epithelial cell line. Shear stress generated by the primary filtrate flow in the proximal tubule was mimicked and adherence of infusing UPEC under these conditions was studied by time-lapse microscopy using model isolate CFT073. The majority of UPEC did not interact with A-498 cells was rapidly cleared. Only a small number of bacteria resisted the flow and was slowly moving in the flow either rolling or weakly attaching to the epithelial cells. Only a few bacteria strongly interacted under shear stress with the epithelial cells.The interaction phenotype of UPEC with A-498 cells was reversible: bound cells can detach again and vice versa. Initially adherent bacteria displayed great fluctuation regarding adherence with a rapid turnover of adherent bacteria. Only a small number of UPEC cells maintained to be strongly attached to the kidney cells and initiated a founder population. Attached bacteria were rapidly dividing and if they attached to A-498 cells for more than 20 min have sufficient time to divide and either shed new bacteria or to expanded three-dimensionally. Such bacterial microcolonies were, despite the shear stress, attached to host cells for longer periods of time (more than one hour) compared to single bacterial cells.

Repetition of these analyses with an isogenic papG double mutant showed that the presence of the P fimbrial adhesin contributes to the duration of bacterial binding to A-498 cells and that bacteria lacking the P fimbrial adhesin were inferior in long-term adhesion under shear stress. The papG deletion mutant was also able to establish microcolonies. Though the duration of bacterial initial binding was shorter, microcolony formation compensated for that and promoted colonization of the kidney cell line in a similar pattern to the wild type.

Deletion of fimH coding for the type 1 fimbrial adhesin did not affect the range of binding duration of single cells or bacteria multiplication. However, cellular colonization by ΔfimH did not involved microcolony formation and rather occurred as a bacterial monolayer directly attaching the epithelial cell surface.

Optotracing of microcolonies of the different mutants tested in this study in the presence of EbbaBiolight 680 did not allow to detect extracellular matrix production in the microcolonies suggesting that extracellular matrix production does not contribute to early microcolony formation stages under these conditions.

This study reports on the synergistic effects that type 1- and P fimbrial expression has during the early steps of urothelial colonization, which is interesting. The idea to use an experimental system that allows to test the effect of shear stress is sensible and should be a good tool to mimick relevant conditions in the proximal tubule.

We thank the reviewer for their extensive analysis of the paper and the positive comments on the paper premise.

Comment 1: As a control, a fimHpapG1papG2 triple deletion mutant needs to be tested in the PToC system. If the authors are right, the triple deletion mutant would be a negative control.

Response: While we appreciate the reviewer’s comment, we believe that the triple mutant would not accurately serve as a negative control. This is because previous genetic analysis of the expression of P and Type 1 fimbriae has shown that an individual E. coli bacterium only express one of the fimbriae at a time due to co-regulation of the operons (Holden and Gally, 2004; Rhen, Makela and Korhonen 1983, Snyder et al 2005), which we also reference in our earlier paper (Melican et al 2011). The synergy we refer to is at a population level, rather than an individual cell level. Thus, we believe that the controls included in the original submission, alongside those added to the revised version in response to this reviewer’s Comments 3 and 4 are sufficient and serve as correct negative controls.

Comment 2: It is interesting that deletion of the type 1 fimbrial adhesin gene affects interbacterial recognition thus promoting microcolony formation. It is known that type 1 fimbria expression contributes to pellicle formation in static cultures, but this reviewer thought that such type 1 fimbria-mediated interactions were due to interactions between the fimbrial filaments (and not due to the action of the fimbrial adhesin). Against this background, a higher resolution analysis of the cell surface exposed structures will be interesting to get an idea which structures may be involved in interbacterial interaction. Are fimbrial filaments produced at all in the mutants and are they involved in this type of interaction?

Response: The work we present in this paper regarding FimH is supported by our previous in vivo data which also demonstrated inter-bacterial binding mediated by the FimH tip adhesin during kidney infection (Melican et al 2011). This role of FimH has also been described by others, of note is detailed work delineating the role of the FimH tip adhesin in bacterial autoaggregation (Schembri, Christiansen and Klemm, 2008) as well as in biofilm formation (Schembri and Klemm, 2001). More recently cryoelectron microscopy has been used to elucidate the structure of the Type 1 fimbria, with FimA subunits shown to provide a structural scaffold for displaying the tip adhesin, FimH (Spaulding et al, eLife 2018).

In the revised manuscript, our finding of a direct role of FimH is further strengthened by addition of new data from experiments suggested in Comment 3 from this Reviewer. As described in our response to Comment 3, we have added an experiment performed in the presence of D-mannose, and found that it blocks microcolony formation. Though we agree that it would be interesting to investigate the expression and structural interactions between fimbriae structures in microcolony formation, this question must be addressed in future work since the required level of visual resolution is not possible in the present model system.

Comment 3: If indeed, FimH-mediated binding to mannosyl residues-containing structures plays a role here, microcolony formation of type 1 fimbriae-expression bacterial should be reduced in the presence of D-Mannose in the interaction medium. This should be tested.

Response: We have added new experiments to the revised manuscript in which D-Mannose has been added into the PToC. The corresponding data, which are shown in the new Supplementary Fig. 6b, further strengthens our finding that FimH of Type 1 fimbriae are directly involved in the formation of bacterial microcolonies.

Changes made to the manuscript

Result, p 11, lines 270- 274: text added

Materials and Methods, p 17, line 425-428: text added

Supplementary Figure 6b: figure showing experimental data

Comment 4: Molecular biology studies always benefit when analysing deletion mutants from the fact that these mutations can be complemented to prove the relevance and specificity of the mutation. It would therefore also be useful and desirable in this study if appropriate complemented mutants were included.

Response: We have now added experiments in which the CFT073 ∆fimH strain has been complemented with a constructed complementation vector pBAD-fimH under an arabinose inducible promoter. This complementation restored the yeast agglutination phenotype and ultimately the microcolony forming phenotype in the PToC. This data further enhances our findings that FimH is directly involved in microcolony formation.

However, we were unfortunately unsuccessful in complementing the CFT073 ΔpapG ΔpapG_2, despite trying two separate vector approaches, using either a pBAD construction or a previously used plasmid (pKTH3020 Melican et al 2011 ). Neither vector was able to fully restore the RBC agglutination phenotype. We believe this is due to the fact CFT073 has two individual PapG operons, both of which have been disrupted in our strain.

Changes made to the manuscript on fimH complementation

Result, p 11, line 269-273: new text added

Materials and Methods, p17, lines 425-428, and p 20, lines 507-514: new text added

Supplementary Figure 6a: figure showing experimental data

Supplementary Tables 1 and 2: Information on strains, plasmids and oligos added

Comment 5: The authors tested the interaction of UPEC strain CFT073 with non-differentiated A-498 epithelical cells. Proximal tubular cells are the most abundant cell type of all renal tubule epithelial cells. Nevertheless, there are also less frequently present cell types, incl. TAL and DCT cells as well as AQP2-expressing cells (CNT, PC, and IMCD) that can be found in the renal epithelium. Some of them have been suggested to be important for interacting with invading UPEC (e.g. intercalating cells). Similarly, there is also shear stress acting on UPEC when they colonized the bladder. Did the authors try to test bladder epithelial cells lines in the PToC system as well or to mix A-498 cells with other renal epithelial cells lines to see whether certain cell types are more relevant to interacting with UPEC than others?

Response: There are indeed a number of cell types in the kidney. In the PToC model we focus on the dominant renal epithelial cell as in our in vivo models this is the cell type we see most interacting with UPEC (Månsson et al 2006, Melican et al 2008, 2011). We have not tried mixing cell types at this stage though it may indeed be of interest in the future (as commented in the final sentence of the Discussion).

Regarding bladder epithelial cells, we know that they work well in the PToC system. However, the shear stress in the bladder fluctuates very dramatically upon voiding, and this fluid flow cannot currently be modelled as accurately as the flow of the renal tubule. We agree, however, that this is certainly an avenue which will be followed up as we continue developing these biomimetic systems.

**Reviews of manuscript FEMSMC-2022-027.r1**

**Reviewer 3 - Mark Schembri, University of Queensland**

**Recommendation: Accept**

I reviewed the revised manuscript. This is an outstanding study of the highest quality. I commend the authors on their excellent work.

**Reviewer 2**

**Recommendation: Accept**

The authors included a few controls to further strengthen their analysis of the contribution of P- and type 1 fimbriae to initial UPEC-host interaction using a proximal-tubule-on-chip (PToC) with A-498 human renal epithelial cells.

Their findings on differential, but synergistic contribution of P- and type 1 fimbriae to the early interaction steps of UPEC with urothelial cells have now been in part validated with complemented mutants at least for the fimH mutant. Unfortunately, no attempt was made to complement the papG mutant and a triple mutant was not tested to further substantiate the importance of these two fimbrial types for the initial interaction. The importance of the FimH adhesin was further confirmed experimentally by also performing the experiments in the presence of D-mannopyranoside.

Overall, additional controls were added to improve the study and at least investigate the role of type 1 fimbriae in the experimental context a little more substantially.
